# Surgical Fixation of Atypical Femur Fractures in Bisphosphonate-treated Patients

**DOI:** 10.7759/cureus.4690

**Published:** 2019-05-17

**Authors:** Irfan Muhammad Rajput, Jagdesh Kumar, Adeel A Siddiqui, Muhammad Jamil, Muhammad Soughat, Malik W Ahmed

**Affiliations:** 1 Orthopaedic Surgery, Dow University of Health Sciences, Karachi, PAK; 2 Orthopaedics, Dow University of Health Sciences, Karachi, PAK; 3 Orthopaedic Surgery, Dow Medical College Karachi, Karachi, PAK

**Keywords:** atypical femoral fracture, bisphosphonate, intramedullay fixation device, proximal femoral nail antirotation

## Abstract

Objective

To evaluate the outcomes of the surgical fixation of atypical femoral fractures in bisphosphonate-treated patients with an intramedullary device.

Materials and methods

This multicentric study was carried out at the department of orthopedics, Dr. Ruth Phau Civil Hospital and Medicare Hospital, Karachi, Pakistan, between 2013 and 2018. In this retrospective observational study, we reviewed 10 bisphosphonate-treated patients, fixed surgically with an intramedullary nail after presenting with radiologically characteristic atypical femur fractures identified according to the American Society for Bone and Mineral Research criteria. We excluded patients with fractures sustained by high-energy trauma, road traffic accidents, fall from a height, and those associated with underlying malignancy.

Results

A total of 11 atypical femoral fractures in 10 patients were included, all of whom were females with a mean age of 68.6 (range 57-82) years. Out of 11 fractures, 81.8% (n=9) were located in the subtrochanteric region and two were located in the femoral shaft. The mean bisphosphonate use was 58.3 months. All patients were treated with intramedullary devices; an intramedullary interlocking nail in two cases and proximal femoral nail antirotation in nine cases. The mean follow-up duration was 12 months. All fractures were united in an average time of 9.9 months (range 6 - 16 months). Implant failure and/or nonunion were not observed, whereas delayed union was noted in five patients.

Conclusion

Intramedullary fixation is a reliable method for the treatment of atypical femur fractures in bisphosphonate-treated patients owing to its intramedullary placement. These devices act as an internal splint and can provide much more axial stability, reducing the risk of implant fatigue failure due to a delay in fracture healing from prolonged bisphosphonate use.

## Introduction

Osteoporosis is the biggest global health issue, affecting millions of people worldwide, with significant morbidity and mortality due to fragile hip fractures in the elderly population [1-2].

Bisphosphonates are the most commonly prescribed anti-resorptive agents for the prevention of hip and vertebral body fractures in elderly osteoporotic patients. Nonetheless, prolonged bisphosphonate therapy may lead to atypical subtrochanteric and diaphyseal femoral fractures [3-5]. However, the true pathogenesis of atypical femur fractures (AFFs) related to bisphosphonates is still unknown.

In 2005, Odvina et al. were the first who reported the possible link between bisphosphonates and AFFs [6]. They suggested that prolonged bisphosphonate therapy may cause severe and prolonged suppression of bone turnover, resulting in an impaired ability of the bone to remodel, eventually leading to an accumulation of microdamage and insufficiency fractures. Since then, several additional reports regarding atypical femur fractures in elderly patients with prolonged use of bisphosphonates have been published [7].

Bisphosphonate-related atypical femur fractures are very rare, with most patients presenting to emergency with a ground-level fall or with prodromal symptoms of inguinal or groin pain. These atypical femur fractures are often atraumatic in nature and radiologically have a transverse or short oblique fracture line with minimal comminution, a medial spike, and focal lateral cortical thickening. Incomplete contralateral fractures and localized thigh pain may be present before preceding the fracture in up to one-half of the cases.

Bisphosphonate-related atypical femur fractures are very difficult to manage and require a great deal of care, due to the long half-life of bisphosphonate therapy (months to years) and the inhibition of bone healing [8]. A multitude of different extra- and intramedullary fixation devices for these subtrochanteric atypical fracture fixation have been suggested. Recently, several studies have evaluated its outcome and reported high complication rates of delayed fracture union, nonunion, and implant failure with extramedullary fixation devises in comparison with intramedullary fixation devices [9].

After considering these drawbacks, we are using intramedullary devices: proximal femoral nail antirotation (PFNA) and intramedullary interlocking nails at our institution for these atypical femoral fractures, although the risk of nonunion and implant fatigue failure seems to be higher than with a typical osteoporotic femoral fracture. The aim of this study was to evaluate the outcomes of surgically fixed AFFs in bisphosphonate-treated patients with an intramedullary fixation device.

## Materials and methods

This multi-center, retrospective analysis was carried out at the orthopedic department of Dr. Ruth Phau Civil Hospital and Medicare Hospital, Karachi, Pakistan, during the period August 2013 to December 2018. In this study, we retrospectively reviewed the medical records and radiographs of 10 bisphosphonate-treated patients, fixed surgically with an intramedullary nail after presenting with radiologically characteristic atypical femur fractures identified according to the American Society for Bone and Mineral Research (ASBMR) criteria [10] (Table 1). Out of these 10 patients, one patient with a bilateral atypical femur fracture in the subtrochanteric region was initially fixed with a proximal femur locking plate somewhere else, arrived in our emergency department with a broken implant after four months of fixation along with a contralateral incomplete atypical subtrochanteric femur fracture. In this patient, both sides were fixed with intramedullary PFNA and bone grafting was done on the implant failure side. We excluded patients with fractures sustained by high-energy trauma, road traffic accidents, and fall from a height, and those associated with underlying malignancy.

**Table 1:** Revised case definition of atypical femoral fractures according to the American Society for Bone and Mineral Research Task Force 2013 criteria

Major Features	Minor Features
Fracture is associated with minimal or no trauma	Generalized increase in cortical thickness of the femoral diaphysis
Fracture line originates at the lateral cortex and is substantially transverse in its orientation, although it may become oblique as it progresses medially across the femur	Unilateral or bilateral prodromal symptoms such as dull or aching pain in the groin or thigh
Completed fractures extend through both cortices and may be associated with a medial spike	Bilateral incomplete or complete femoral diaphyseal fractures.
The fracture is non-comminuted or minimally comminuted.	Delayed fracture healing.
Incomplete fractures involve only the lateral cortex	Localized periosteal or endosteal thickening of the lateral cortex is present at the fracture site (breaking or flaring)

Prefixation radiographs of the pelvis and affected hip with femur were reviewed and ASBMR criteria were used to diagnose AFFs. Before fracture fixation, bisphosphonate therapy was held. Fracture fixation was undertaken on an elective list under spinal/epidural anesthesia in a supine position on an orthopedic traction table under C-arm control. An intramedullary fixation device was used in all cases, with standard surgical guidelines.

Predesigned data collection sheets were used. The patients’ demographics and clinical data, including age, sex, fracture location (subtrochanteric or diaphyseal), fixation method, time to union, postfixation complications (delayed union, nonunion, implant fatigue failure), duration of bisphosphonate therapy, need for revision surgery, and follow-up duration, were gathered through a review of medical record and radiographs.

The fractures were labeled as 'healed' radiologically if they showed a bridging callus in two orthogonal views and clinically if the fracture site was nontender and pain-free on weight bearing.

## Results

**Figure 1:**
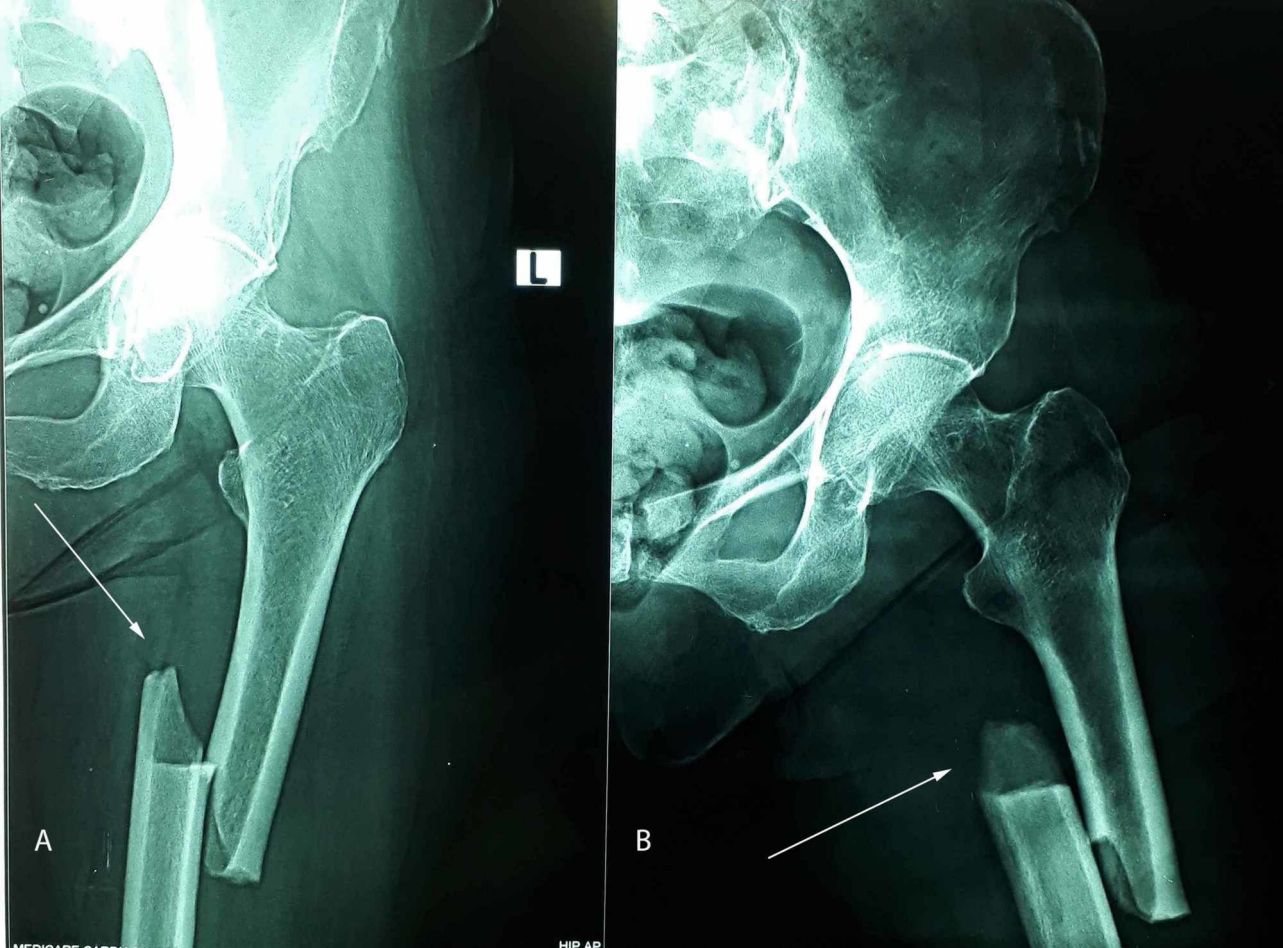
Preoperative anteroposterior (1A) and lateral view (1B) X-ray of a 61-year-old female showing features of atypical diaphyseal femoral fracture

**Figure 2:**
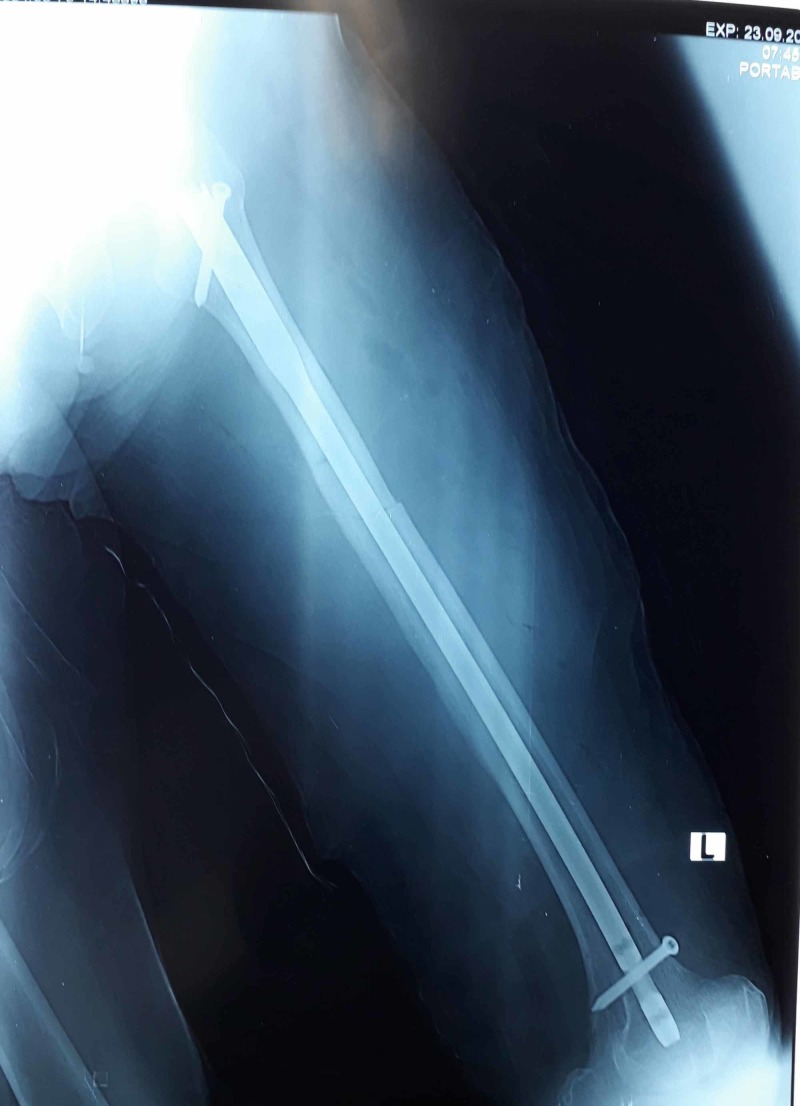
Postoperative X-ray of patient showing fixation with intramedullary interlocking nail

**Figure 3:**
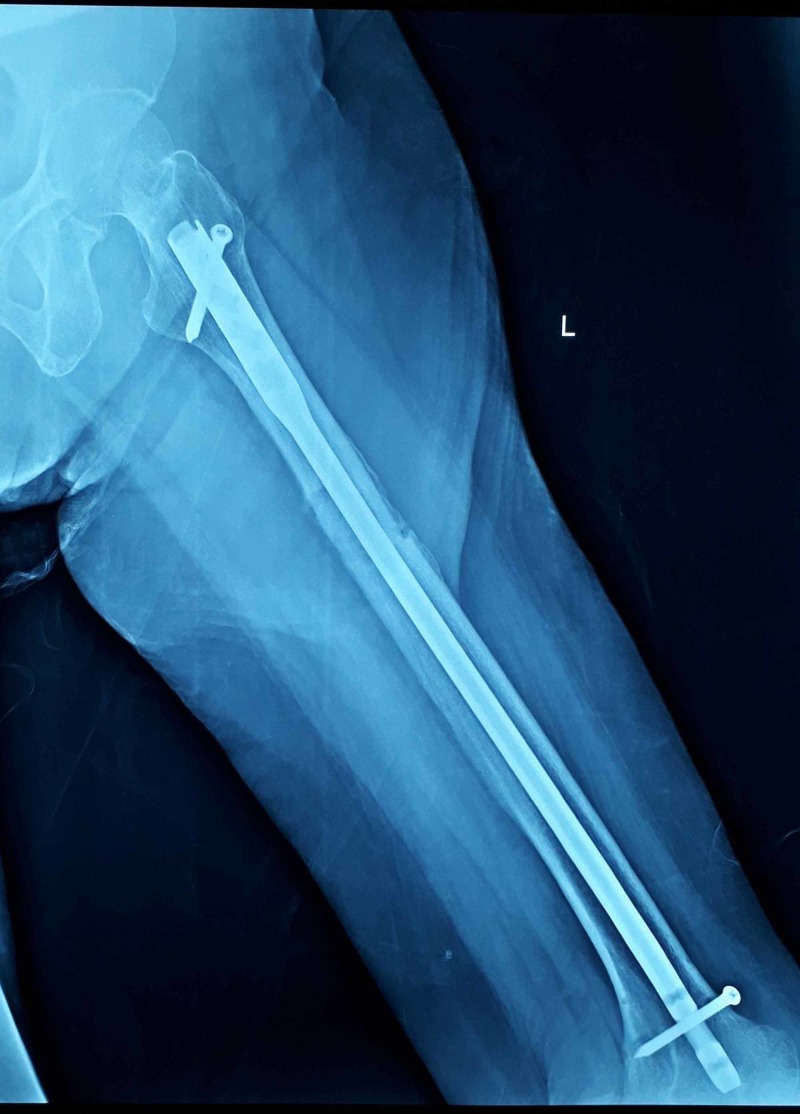
Postoperative lateral view X-ray after eighth month of intramedullary fixation with interlocking nail showing good fracture healing

**Figure 4:**
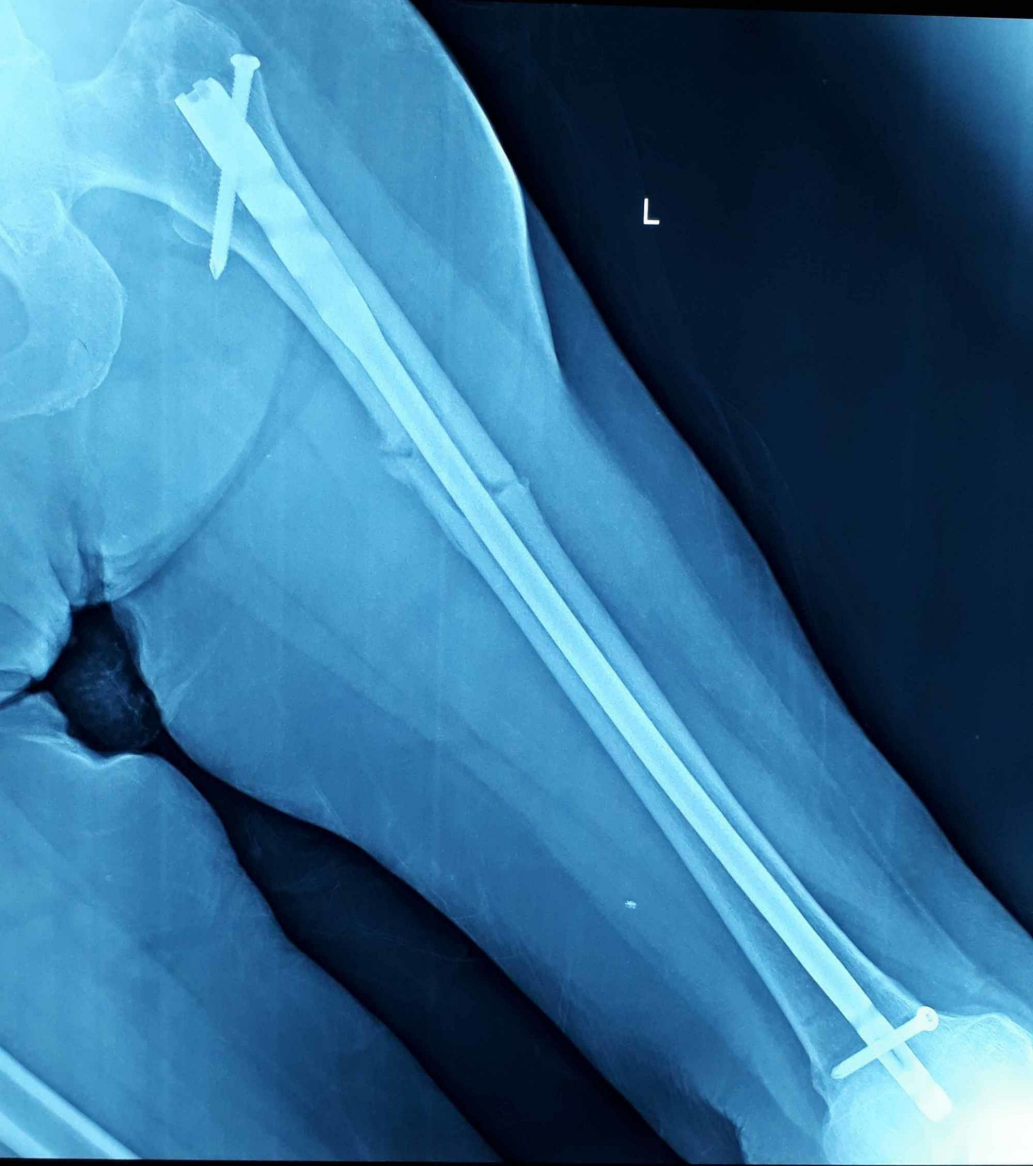
Postoperative X-ray anteroposterior view after eighth month of intramedullary fixation with interlocking nail showing good fracture healing

As shown in Table 2, a total of 11 atypical femoral fractures in 10 patients were included, all of whom were females with a mean age of 68.6 (range 57-82) years. All patients had experienced the fracture from a ground-level fall. A bilateral fracture was noted in one case. Out of 11 fractures, 81.8% (n=9) were located in the subtrochanteric region and two were located in the femoral shaft, one of which can be seen in Figure 1. Of the 10 patients, four were taking alendronate for the treatment of osteoporosis before their fracture, whereas ibandronate and zoledronate were taken by three patients each. The mean bisphosphonate use was 58.3 months (range 42-80 months). All patients were treated with intramedullary devices; an intramedullary interlocking nail in two cases and PFNA in nine cases. The mean follow-up duration was 12 months (range 9 to 16 months). All fractures were united in an average time of 9.9 months (range 6 - 16 months). Some examples are shown in Figures 2-4. Implant failure and/or nonunion were not observed whereas delayed union was noted in five patients. All progressed to union with an additional minor surgical procedure; bone grafting in three cases, bone grafting and bone morphogenetic proteins in one, and dynamization in one case.

**Table 2:** Demography of patients with atypical femoral fractures Abbreviation: PFNA, proximal femoral nail antirotation; IM, Intramedullary.

Serial Number	Age (years)	Gender	Fracture location	Fracture side (Right/Left)	Fixation method	Type of Bisphosphonates	Duration of Bisphosphonates (months)	Time of union (months)
1	69	Female	Subtrochanteric	Left	PFNA	Alendronate	72	16
			Stress fracture Subtrochanteric	Right	Expert IM nail			12
2	65	Female	Subtrochanteric	Left	PFNA	Zoledronic Acid	48.2	8
3	74	Female	Subtrochanteric	Left	PFNA	Zoledronic Acid	60	11
4	73	Female	Subtrochanteric	Right	PFNA	Alendronate	60	14
5	78	Female	Diaphyseal	Left	PFNA	Zoledronic Acid	50.6	7
6	62	Female	Subtrochanter	Right	PFNA	Ibandronate	66.6	8
7	57	Female	Subtrochanter	Left	PFNA	Alendronate	48	6
8	82	Female	Subtrochanter	Right	PFNA	Ibandronate	80	13
9	65	Female	Subtrochanteric	Left	PFNA	Alendronate	56	6
10	61	Female	Diaphyseal	Left	IM nail	Ibandronate	42	8

## Discussion

With an increasingly aging population, the use of anti-osteoporotic agents is on a gradual rise and atypical femur fractures related to bisphosphonate administration are being reported. Now, many researchers show that prolonged bisphosphonate administration may lead to a higher incidence of AFFs [3,11].

Surgical fixation of bisphosphonate-related AFFs is still a matter of debate; Recently, several studies have evaluated its outcome and reported that atypical femur fractures associated with bisphosphonates had higher rates of postoperative implant fatigue failure with extramedullary plate osteosynthesis, due to a delay in fracture healing from prolonged bisphosphonate use [8].

After considering these drawbacks, we are using intramedullary devices: PFNA and interlocking nails at our institution for these subtrochanteric and diaphyseal AFFs.

Several studies have looked at its outcomes and reported reduced implant fatigue failure due to a delay in union in comparison with plate fixation [12-13].

Prasarn et al. conducted a retrospective comparative study, which reported that AFFs associated with bisphosphonates had higher rates of postoperative plate failure (30%) compared with similar fractures not treated with bisphosphonates (0%), although implant failure did not occur in atypical femur fractures treated by an intramedullary interlocking nail [12].

Similarly, Teo et al. [9] retrospectively reviewed 33 consecutive female patients, who were treated surgically for atypical femur fractures and reported that the rate of implant failure and revision rate were much higher in the extramedullary device group (29% and 38%) than in the intramedullary device group (11.1% and 22.2%). He believed extramedullary devices underwent implant fatigue failure due to a delay in union. In addition, intramedullary devices are weight-shearing implants that allow immediate weight-bearing even in unstable fractures.

Bogdon Y et al. also did a multicenter retrospective review of 179 patients operatively treated for atypical femur fractures with intramedullary devices and reported a 12% failure rate and a delayed average time to union [8].

Egol et al. treated 33 patients with 41 atypical femur fractures associated with ≥ 5 years of bisphosphonate use and reported a high rate of union with intramedullary nailing; 98% of fractures ultimately united at a mean of 8.3 months but had taken a longer healing time than that for a typical femur fracture [2].

In our study, all fractures were united in an average time of 9.9 months (range 6-16 months). However, 45% (5 cases) did not show union within eight months after surgery, but all progressed to union with an additional minor surgical procedure: bone grafting in three cases, bone grafting and bone morphogenic proteins in one, and dynamization in one case. There was no implant failure and/or nonunion in our study. All patients had also received bisphosphonate therapy for more than 36 months and the mean duration was 58.3 months.

In our study, we only prescribed calcium and vitamin D supplements after the discontinuation of bisphosphonates therapy. We did not use teriparatide treatment for the reversal of the bisphosphonate effect because evidence is still lacking. Some reports suggest their use in bone healing, but an actual comparison with or without teriparatide treatment is not yet available [14]. Fortunately for our patient, we were still able to attain union by only the fixation of the fracture site with intramedullary devices.

The limitations of this paper include its small sample size and retrospective study design. Also, a lack of comparison between extramedullary and intramedullary fixation devices is a deficiency. Still, we believe it will provide benefits to orthopedic surgeons treating atypical femur fractures.

## Conclusions

Our study and other published studies suggest that intramedullary fixation is a reliable method for the treatment of atypical femur fractures in bisphosphonate-treated patients owing to its intramedullary placement. These devices act as an internal splint and can provide much more axial stability thus reducing the risk of implant fatigue fracture due to a delay in fracture healing from prolonged bisphosphonate use.
